# Salicylic Acid; How Does It Cure Rheumatism

**Published:** 1887-04

**Authors:** 


**Salicylic Acid ; How does it Cure Rheumatism ?**—Dr. T. Davis Pryce (in the *London Lancet*) states that there is good reason to believe that the salicylates act by destroying uric ac d. He says : I obtained the urine of a healthy man, and from this uric acid was readily thrown down by the ordinary method. From the same person under similar circumstances as to time, food, etc., but to whom salicylate of soda had been administered, I procured another sample of urine. In this instance uric acid could not be obtained, no trace of it being discernable by the murexide test. Further experiments of this kind did not uniformly present the same results, but in all cases the amount of uric acid found in the urine containing salicyluric acid was very appreciably diminished. I may add that subsequent clinical experience has strengthened my belief in the above property of salicylic acid and the salicylates.

